# Targeting Grade-Specific Endoplasmic Reticulum Stress Vulnerabilities in Chondrosarcoma: Divergent Roles of Protein Kinase R-Like Endoplasmic Reticulum Kinase and Inositol-Requiring Enzyme 1α Signaling

**DOI:** 10.34133/cancomm.0026

**Published:** 2026-05-14

**Authors:** Hongtai Chen, Zezhuo Su, Ying Lee Lam, Raymond Ching Hin Yau, Anderson Siu Ming Leung, Gabriel Ching Ngai Leung, John Robert Honiball, Richard O. C. Oreffo, Jason Pui Yin Cheung, Siu Wai Choi, Kelvin Sin Chi Cheung

**Affiliations:** ^1^ Department of Orthopaedics and Traumatology, Li Ka Shing Faculty of Medicine, The University of Hong Kong, Hong Kong SAR, P. R. China.; ^2^ Department of Orthopaedics and Traumatology, Shenzhen Hospital of Shanghai University of Traditional Chinese Medicine, Shenzhen, Guangdong, P. R. China.; ^3^ Orthopaedic Oncology and Limb Salvage Surgery, Department of Orthopaedics and Traumatology, Queen Mary Hospital, Hong Kong SAR, P. R. China.; ^4^ Department of Orthopaedics and Traumatology, Hong Kong Sanatorium and Hospital, Hong Kong SAR, P. R. China.; ^5^ Bone and Joint Research Group, Centre for Human Development, Stem Cells and Regeneration, Institute of Developmental Sciences, University of Southampton, Southampton, UK.

Chondrosarcoma (CHS) is the second most common primary skeletal malignancy and remains challenging to treat due to radio-chemoresistance [1,2]. We previously established a single-cell transcriptomic atlas of CHS, identifying increased expression of endoplasmic reticulum (ER) stress-related genes during progression from benign chondroid lesions to low-grade CHS (LCHS) [3]. In the study, we aimed to investigate the effects of ER stress inducer HA15 [4] and inhibitors ISRIB [5] and 4μ8C [6] in LCHS and high-grade CHS (HCHS), using patient-derived tissues and corresponding primary cells from 3 samples per grade in vitro and in vivo (Tables S1 and S2). Materials and methods are detailed in the Supplementary Materials.

Histologically, LCHS preserved cartilage-like features with proteoglycan production, whereas HCHS exhibited malignant features including glycosaminoglycan depletion, high cellularity, and increased mitotic activity (Fig. S1). Gene expression analysis revealed up-regulated ER-stress-related genes in LCHS, with activation of the protein kinase R-like ER kinase (PERK) pathway. In contrast, HCHS showed up-regulation of inositol-requiring enzyme 1α (IRE1α) pathway-related genes, indicating activation of the prosurvival signaling pathway (Fig. S2). In vitro, HCHS cells possessed higher proliferation, migration, and invasion compared to LCHS cells (S phase, 26.34% versus 13.17%; healing, 82.86% versus 52.39%; invasion, 124 versus 29 cells; Fig. 1A and Fig. S3).

**Fig. 1.**
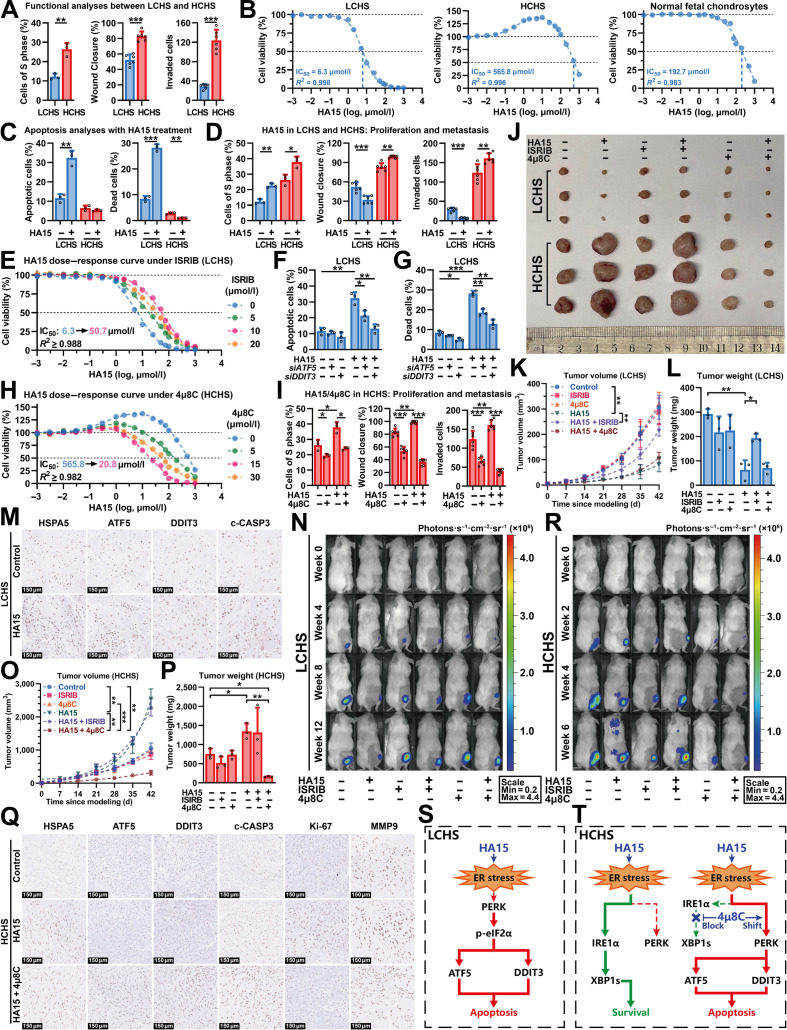
Patient-derived xenograft models revealed grade-specific endoplasmic reticulum (ER) stress vulnerabilities and potential therapeutic strategies in chondrosarcoma. (A) Comparison between low-grade chondrosarcoma (LCHS) and high-grade chondrosarcoma (HCHS) cells via​ cell cycle analysis (S phase percentage) using flow cytometry with FxCycle Violet and 5-ethynyl-2′-deoxyuridine (EdU) staining (*n* = 3; unpaired *t* test; left), wound healing assay measuring closure after 48 h in 2% serum (*n* = 6; unpaired *t* test; middle), and​ a 48-h Transwell invasion assay using Matrigel-coated inserts (*n* = 6; unpaired *t* test; right). (B) Cell viability dose–response curves for LCHS (left), HCHS (middle), and normal fetal chondrocytes (right) following 48-h treatment with increasing concentrations of HA15 (*n* = 6 per group). (C and D) Analysis of LCHS and HCHS cells treated with HA15 (20 μmol/l) for 48 h. The apoptosis and cell death percentages were assessed by flow cytometry with annexin V/propidium iodide (PI) staining (*n* = 3; unpaired *t* test) (C), and S phase cell proportion was determined by cell cycle analysis (*n* = 3; unpaired *t* test), and migration and invasion were evaluated by measuring wound closure in a scratch assay and a Matrigel-based Transwell assay (*n* = 6; unpaired *t* test) (D). (E) Cell viability dose-response curves of LCHS cells with increasing HA15 concentrations after 48-h treatment with ISRIB in various concentrations (0, 5, 10, and 20 μmol/l; *n* = 6). (F and G) Quantification of apoptotic (F) and dead (G) LCHS cells by flow cytometry with Annexin V/PI staining after 48-h treatment with or without HA15 (20 μmol/l), in the presence or absence of ATF5 or DDIT3 knockdown (*n* = 3; one-way analysis of variance [ANOVA] test). (H) Cell viability dose–response curves of HCHS cells with increasing HA15 concentrations after 48-h treatment with 4μ8C in various concentrations (0, 5, 15, and 30 μmol/l; *n* = 6). (I) Effect of HA15 and 4μ8C on cell cycle progression, migration, and invasion in HCHS cells. The percentage of cells in S phase determined by cell cycle analysis (left), wound closure measured by scratch assay (middle), and the number of invaded cells determined by Matrigel-based Transwell assay (right) were assessed in HCHS cells treated with HA15 (20 μmol/l) in the presence or absence of 4μ8C (15 μmol/l) for 48 h (*n* = 6; unpaired *t* test). (J to L) Subcutaneous patient-derived xenograft (PDX) tumors of LCHS and HCHS that harvested from mice after treatment with vehicle, HA15 (20 mg/kg, intraperitoneally [ip], every other day [qod]), ISRIB (2.5 mg/kg, ip, qod), 4μ8C (20 mg/kg, ip, qod), or their combination (*n* = 3 per group). The images of tumors harvested from mice in the indicated groups (J). The curves of real-time tumor volume measured over 42 d since modeling (K) and the final tumor weights at end point (L) (one-way ANOVA test). (M) Representative immunohistochemical staining for heat shock protein family A member 5 (HSPA5), activating transcription factor 5 (ATF5), DNA damage-inducible transcript 3 (DDIT3), and cleaved caspase-3 (c-CASP3) in LCHS tumor sections harvested from mice after treatment with vehicle or HA15 (20 mg/kg, ip, qod). (N)​ Bioluminescence imaging of intraosseous LCHS PDX models treated with vehicle, HA15 (20 mg/kg, ip, qod), ISRIB (2.5 mg/kg, ip, qod), 4μ8C (20 mg/kg, ip, qod), or their combinations over 12 weeks (*n* = 3 per group). (O and P) In vivo analysis of subcutaneous HCHS PDX models treated with vehicle, HA15 (20 mg/kg, ip, qod), ISRIB (2.5 mg/kg, ip, qod), 4μ8C (20 mg/kg, ip, qod), or their combinations. The curves of real-time tumor volume measured over 42 d since modeling (O) and the final tumor weights at end point (P) (one-way ANOVA test). (Q) Representative immunohistochemical staining images for HSPA5, ATF5, DDIT3, c-CASP3, Ki-67 (marker of proliferation Ki-67), and matrix metallopeptidase 9 (MMP9) in HCHS tumor sections. (R) Bioluminescence imaging of intraosseous HCHS PDX models treated with vehicle, HA15 (20 mg/kg, ip, qod), ISRIB (2.5 mg/kg, ip, qod), 4μ8C (20 mg/kg, ip, qod), or their combinations over 6 weeks (*n* = 3 per group). (S) Schematic diagram illustrating the proposed mechanism in LCHS, wherein HA15 induces ER stress, leading to sequential activation of protein kinase R-like ER kinase (PERK), phosphorylated eukaryotic initiation factor 2α (p-eIF2α), and subsequent induction of ATF5 and DDIT3, culminating in apoptosis. (T) Schematic diagram illustrating the proposed ER stress response pathways in HCHS cells: The left panel depicts HA15-induced ER stress signaling through inositol-requiring enzyme 1α (IRE1α) and X-box binding protein 1 splicing (XBP1s), and the right panel depicts the proposed mechanistic shift upon combined treatment with HA15 and IRE1α inhibitor 4μ8C, which blocks XBP1s splicing and is associated with increased PERK pathway signaling. In all panels, blue and red bars represent LCHS and HCHS, respectively; statistical significance is denoted by asterisks (*P < 0.05, **P < 0.01, and ***P < 0.001).

The cellular response to ER stress is determined by both its intensity and duration [7]. Sustained low-level ER stress may promote tumorigenic processes including immunosuppression and radio-chemoresistance, whereas severe ER stress triggers DNA damage-inducible transcript 3 (DDIT3)-mediated apoptosis [7]. To investigate the effect of ER stress on different grades of CHS, we induced ER stress in LCHS and HCHS cells with HA15, an inhibitor of heat shock protein family A member 5 (HSPA5) [4]. LCHS cells, with a high baseline level of ER stress [3], were found to be highly sensitive to HA15, with a half-maximal inhibitory concentration (IC_50_) of 6.3 μmol/l. In comparison, HCHS cells demonstrated resistance to HA15 treatment with an IC_50_ of 565.8 μmol/l. As control, normal chondrocytes derived from the fetal femur showed intermediate sensitivity with an IC_50_ of 192.7 μmol/l (Fig. 1B). Functionally, HA15 induced apoptosis in LCHS cells (Fig. 1C and Fig. S4). Critically, HA15-treated HCHS cells showed substantial increased proliferation, migration, and invasion (Fig. 1D and Fig. S5), demonstrating CHS grade-specific response to ER stress.

In LCHS, we previously observed PERK pathway activation and up-regulation of activating transcription factor 5 (ATF5) and DDIT3 [3]. As these are established mediators of apoptosis [8], we further investigated the response to HA15-induced ER stress and found that it up-regulated PERK-pathway-associated genes and proapoptotic markers (Fig. S6). To confirm PERK pathway dependency in LCHS, we used ISRIB, which reverses PERK-mediated eukaryotic translation initiation factor 2 α (eIF2α) phosphorylation [5]. Concurrent treatment with ISRIB (10 μmol/l) elevated the IC_50_ of HA15 from 6.3 to 50.7 μmol/l in LCHS cells (Fig. 1E). This protective effect was consistent with a reduction in HA15-mediated proapoptotic gene expression (Fig. S6). Furthermore, ATF5 or DDIT3 knockdown in LCHS using small interfering RNA significantly inhibited HA15-induced apoptosis (Fig. 1F and G and Fig. S7). Together, these findings demonstrated that PERK signaling promotes apoptosis via ATF5 and DDIT3 during ER stress response in LCHS.

In HCHS, higher IRE1α pathway activation was observed compared to LCHS (Fig. S2). IRE1α pathway activation generates spliced X-box binding protein 1 (XBP1s), a key transcription factor that promotes cell survival [9]. We hypothesized that XBP1s-induced up-regulation of downstream genes may underlie HA15 resistance and enhance proliferation, migration, and invasion in HCHS cells under ER stress (Fig. S8). To investigate the importance of the IRE1α pathway in HCHS, we used 4μ8C (15 μmol/l), an IRE1α/XBP1s inhibitor. Our data demonstrated that treatment with 4μ8C sensitized HCHS to HA15, reducing IC_50_ of HA15 from 565.8 to 20.8 μmol/l, a value lower than that for normal chondrocytes and within the therapeutic window (Fig. 1H). Gene expression analysis suggested that 4μ8C treatment redirected HA15-induced ER stress signaling from prosurvival IRE1α/XBP1s pathway toward proapoptotic PERK pathway in HCHS (Fig. S8). Functionally, HA15 + 4μ8C combination treatment reduced proliferation, migration, and invasion in HCHS cells compared to HA15 monotherapy (Fig. 1I and Fig. S9), indicating that IRE1α/XBP1s inhibition switches the ER stress response from prosurvival to apoptosis in HCHS.

To evaluate the efficacy of HA15 and 4μ8C treatment for CHS in vivo, we used subcutaneous and intraosseous patient-derived xenograft (PDX) models (Table S1). Subcutaneous PDX models recapitulated CHS subtype growth patterns, with LCHS growing indolently versus rapid HCHS growth (Fig. 1J).

In LCHS, HA15 monotherapy induced tumor regression compared to control, reducing tumor volume and weight at 6 weeks (Fig. 1K and L), with up-regulation of apoptosis markers HSPA5, ATF5, DDIT3, and cleaved caspase-3 (c-CASP3; Fig. 1M and Fig. S10). Intraosseous PDX models further demonstrated HA15 efficacy, showing a marked reduction in bioluminescence compared to control at 12 weeks (Fig. 1N and Fig. S11). The HA15 + ISRIB combination abolished the therapeutic effect of HA15 in LCHS (Fig. 1K, L, and N and Fig. S11), demonstrating PERK pathway dependency of HA15-induced apoptosis in LCHS in vivo.

In HCHS, HA15 monotherapy promoted tumor growth compared to control at 6 weeks, whereas HA15 + 4μ8C combination treatment markedly reduced volume and weight compared to HA15 monotherapy (Fig. 1J, O, and P). Gene expression analysis demonstrated HA15 + 4μ8C combination up-regulated apoptotic markers (ATF5, DDIT3, and c-CASP3) and suppressed proliferation marker Ki-67 and metastasis marker matrix metalloproteinase 9 (MMP9) compared to HA15 monotherapy (Fig. 1Q and Fig. S12). In intraosseous PDX models at 6 weeks, HA15 monotherapy in HCHS increased bioluminescence and metastasis, whereas HA15 + 4μ8C combination reduced both bioluminescence and metastasis (Fig. 1R and Fig. S13). In addition, HA15 + 4μ8C combination therapy extended median survival compared to control from 9.57 to 13.00 weeks (hazard ratio = 0.23, *P* = 0.041; Fig. S14). Together, these results suggested that simultaneous intensification of ER stress and blockade of the IRE1α pathway could serve as an effective treatment in HCHS.

In conclusion, this study uncovers grade-specific ER stress vulnerabilities in CHS. In LCHS, HA15-induced ER stress activates the proapoptotic PERK/ATF5/DDIT3 axis, driving tumor regression in vivo (Fig. 1S). Conversely, HCHS exploits ER stress to promote survival and metastasis through IRE1α/XBP1s signaling. Using HA15 and 4μ8C in combination, we overcome the adaptive ER stress resistance in HCHS by simultaneously intensifying ER stress and redirecting the response from prosurvival to apoptosis, thereby suppressing tumor growth, reducing metastasis, and improving survival (Fig. 1T). Together, our findings establish a potential therapeutic strategy for this recalcitrant malignancy.

This study demonstrates translational potential in targeting grade-specific ER stress pathways in CHS. However, a few limitations should be acknowledged to guide clinical translation. Clinical applicability is limited because of the use of research-grade compounds HA15 and 4μ8C. Furthermore, the roles of key mediators such as reactive oxygen species and metabolic reprogramming remain unexplored. An immediate future direction is to substitute HA15 and 4μ8C with Food and Drug Administration-approved agents with similar cellular effects. This will enable large-scale validation in PDX cohorts and accelerate clinical trials. Furthermore, ER stress signatures (ATF5 and DDIT3) may serve as potential biomarkers for stratification, guiding grade-specific therapeutic strategies. Future efforts should focus on identifying clinically approved agents, elucidating additional mechanisms, and establishing reliable biomarkers to advance this therapeutic strategy for CHS.

## Ethical Approval

This study involving human participants was approved by the Institutional Review Board of the University of Hong Kong/Hospital Authority Hong Kong West Cluster (HKU/HA HKW IRB: UW 16-2036 and UW 21-680). Written informed consent was obtained from all participants prior to inclusion in the study. All animal procedures were approved by the Committee on the Use of Live Animals in Teaching and Research of the University of Hong Kong (CULATR: 23-371) and were performed in accordance with the institutional guidelines.

## Data Availability

The data that support the findings of this study are available within the article and the Supplementary Materials. Further information and requests for resources and reagents should be directed to and will be fulfilled by the lead contact, K.S.C.C. (kc81@hku.hk).
