# Supplementary material for: Targeting Grade-Specific Endoplasmic Reticulum Stress Vulnerabilities in Chondrosarcoma: Divergent Roles of Protein Kinase R-Like Endoplasmic Reticulum Kinase and Inositol-Requiring Enzyme 1α Signaling
Source: Cancer Commun (Lond). 2026 May 14;46:0026. doi: 10.34133/cancomm.0026 (PMC13172731; doi:10.34133/cancomm.0026)
Supplement: Supplementary 1 — Figs. S1 to S14 Tables S1 to S5 [file cancomm.0026.f1.pdf]

Supplementary Materials for

Targeting Grade-specific ER Stress Vulnerabilities in Chondrosarcoma: Divergent Roles of PERK and IRE1α Signaling

Hongtai Chen[1,2,†], Zezhuo Su[1,†], Ying Lee Lam[3,4], Raymond Ching Hin Yau[3], Anderson Siu Ming Leung[3], Gabriel Ching Ngai Leung[3], John Robert Honiball[1], Richard OC Oreffo[5], Jason Pui Yin Cheung[1], Siu Wai Choi[1,*], Kelvin Sin Chi Cheung[1,*]

Affiliations

[1] Department of Orthopaedics and Traumatology, Li Ka Shing Faculty of Medicine, The University of Hong Kong, Hong Kong SAR, P. R. China

[2] Department of Orthopaedics and Traumatology, Shenzhen Hospital of Shanghai University of Traditional Chinese Medicine, Shenzhen, Guangdong, P. R. China

[3] Orthopaedic Oncology and Limb Salvage Surgery, Department of Orthopaedics and Traumatology, Queen Mary Hospital, Hong Kong SAR, P. R. China

[4] Department of Orthopaedics and Traumatology, Hong Kong Sanatorium & Hospital, Hong Kong SAR, P. R. China

[5] Bone and Joint Research Group, Centre for Human Development, Stem Cells and Regeneration, Institute of Developmental Sciences, University of Southampton, Southampton, United Kingdom

[†] Hongtai Chen and Zezhuo Su contributed equally to this work.

[*] **Corresponding author**

Kelvin Sin Chi Cheung: kc81@hku.hk

Siu Wai Choi: htswchoi@hku.hk

Supplementary materials and methods

Patient samples and primary cell culture

Chondrosarcoma tissues were collected from surgical resection, while normal cartilage tissues were obtained from aborted fetuses. Both types of specimens were acquired from Queen Mary Hospital with institutional ethics approval and patient-informed consent (IRB: UW 16-2036 and UW 21-680). Pathologists categorized samples as low-grade (LCHS) or high-grade chondrosarcoma (HCHS), with three independent patient samples for each grade (**Supplementary Table S1**). Fresh samples were minced (<1 mm^3), digested at 37°C for 2 h in 0.4% collagenase II (Gibco, Cat. #17101015), hyaluronidase (Sigma, Cat. #H3384), and dispase II (Sigma, Cat. #D4693), passed through a 70 µm cell strainer (Corning, Cat. #431751), centrifuged (300 ×*g*, 5 min), and resuspended in Dulbecco's Modified Eagle Medium (DMEM; Gibco, Cat. #12100046) supplemented with 10% Fetal Bovine Serum (FBS; Gibco, Cat. #A5256701), 100 U/mL penicillin–streptomycin (Gibco, Cat. #15140122), and 1 µg/mL amphotericin B (Gibco, Cat. #15290018). Cultures were maintained at 37°C in 5% CO_2, with medium changes every 2–3 days. Cells were cryopreserved in FBS with 10% dimethyl sulfoxide (DMSO, Sigma, Cat. #D2650). A portion of each original tumor sample was also formalin-fixed, paraffin-embedded, or snap-frozen and reserved for subsequent experiments.

Histological analysis

Tumors were fixed in 4% paraformaldehyde (4°C, 24 h), paraffin-embedded, and sectioned at 5 µm. For morphological assessment, hematoxylin and eosin (H&E; Abcam, Cat. #ab245880) staining was performed by deparaffinizing sections in xylene, rehydrating them through a graded ethanol series, staining with Harris hematoxylin for 5 minutes, rinsing, differentiating in 1% acid alcohol, bluing, and counterstaining with eosin. To evaluate matrix components, sections were first stained with 0.1% safranin-O (Sigma, Cat. #S2255) for 2 minutes, rinsed, and rapidly differentiated in 50%, 70%, and 80% ethanol; this was followed by counterstaining with 0.05% fast green (Sigma, Cat. #F7258) for 6-30 seconds and rapid dehydration in absolute ethanol. Similarly, glycosaminoglycans were detected using alcian blue (Sigma, Cat. #TMS-010) staining at pH 2.5 for 30 minutes. For all stains, images were captured under light microscopy and quantified for staining intensity to correlate with tumor grade.

Cell viability assay

Chondrosarcoma cells were seeded in 96-well plates (5×10^4 cells/mL, 100 µL/well) and co-treated with HA15 (0.001-1,000 µmol/L), ISRIB (10 µmol/L), and 4µ8C (15 µmol/L) for 48 h (*n* = 6 per group; **Supplementary Table S2**). After drug exposure, 10 µL CCK-8 (Dojindo, Cat. #CK04-05) was added and incubated for 4 h. Absorbance was measured at 450 nm. Cell viability (%) was calculated as a percentage of the control using the formula: (Mean absorbance/fluorescence of treated cells)/(Mean absorbance/fluorescence of control cells); Half maximal inhibitory concentration (IC_{50}) values were derived using GraphPad Prism (version 8, Dotmatics).

Apoptosis analysis

Chondrosarcoma cells were seeded in 12-well plates (1×10^5 cells/mL, 2 mL/well) and transfected to target activating transcription factor 5 (ATF5) and DNA damage-inducible transcript 3 (DDIT3) with *siATF5* and *siDDIT3*, respectively (**Supplementary Table S3**) by Lipofectamine™ RNAiMAX (Thermo Fisher, Cat. #13778030), using 30 pmol siRNA and 9 µL RNAiMAX in Opti MEM (Gibco, Cat. #31985070) for 48 h (*n* = 3 per group). The transfected cells were treated with 20 µmol/L HA15 for 48 h. Both floating and adherent cells were collected, washed with cold PBS, and resuspended in 1× Annexin-binding buffer (Invitrogen, Cat. #V13246) at a density of approximately 1×10^6 cells/mL. Using the Dead Cell Apoptosis Kit (Invitrogen, Cat. #V13242), 5 µL of Annexin V conjugate and 1 µL of a 100 µg/mL propidium iodide (PI) working solution were added to 100 µL of cell suspension, followed by incubation at room temperature for 15 minutes, protected from light. Subsequently, 400 µL of 1× Annexin-binding buffer was added to each sample. The samples were then analyzed on the NovoCyte Advanteon Flow Cytometer Systems (Agilent, Cat. #150217), collecting data for at least 10,000 events per sample using the appropriate channels for Annexin V and PI detection as per the manufacturer's instructions. Data analysis was performed to create dot plots, with quadrants set to distinguish viable cells (Annexin V-negative, PI-negative), early apoptotic cells (Annexin V-positive, PI-negative), late apoptotic/necrotic cells (Annexin V-positive, PI-positive), and necrotic cells (Annexin V-negative, PI-positive). The percentage of

cells in each quadrant was calculated.

Cycle analysis

Chondrosarcoma cells were seeded in 12-well plates (2×10^5 cells/mL, 2 mL/well) and co-treated with HA15 (20 μmol/L), ISRIB (10 μmol/L), and 4μ8C (15 μmol/L) for 48 h ($n = 3$ per group), after which the cells were collected by trypsinization, washed with PBS, and suspended in culture medium. The cells were counted and aliquoted at a concentration of 1×10^6 cells per sample. For EdU labeling (Invitrogen, Cat. #C10632), EdU (5-ethynyl-2'-deoxyuridine) was added to the cell culture medium at a final concentration of 10 μmol/L, and the cells were incubated for 2 h under normal growth conditions. For fixation and permeabilization, the cells were washed once with 3 mL of 1% bovine serum albumin (BSA) in PBS, pelleted, and the supernatant was removed. The cells were then fixed by adding 100 μL of Click-iT® fixative (Invitrogen, Cat. #C10632) and incubating for 15 minutes at room temperature while protected from light. After a wash with 3 mL of 1% BSA in PBS, the cell pellet was dislodged and resuspended in 100 μL of 1× Click-iT® saponin-based permeabilization and wash reagent for a 15-minute incubation. For the Click-iT® reaction, 0.5 mL of Click-iT® reaction cocktail was added, and the cells were incubated for 30 minutes at room temperature in the dark, followed by a wash with 3 mL of the permeabilization and wash reagent. To stain for DNA content, the cells were resuspended in 500 μL of the permeabilization and wash reagent, and 1 μL of FxCycle™ Violet stain (Invitrogen, Cat. #F10347) was added, followed by a 30-minute incubation at room temperature in the dark. The samples were then analyzed on the NovoCyte Advanteon Flow Cytometer Systems (Agilent, Cat. #150217), which was set up according to the manufacturer's instructions. Instrument settings were adjusted using unstained and single-stained controls, and at least 10,000 events were collected per sample using the appropriate channels for EdU and DNA content. Data analysis was performed to create dot plots and histograms. The populations were gated to determine the percentage of cells in G_0/G_1, S, and G_2/M phases, and EdU incorporation was analyzed to assess actively proliferating cells.

Wound healing assay

Chondrosarcoma cells were seeded in 12-well plates at a density of 2×10^5 cells/mL, with 2

mL added to each well. After the cells reached confluence, a straight scratch was made in the monolayer using a 100 μL pipette tip. The monolayer was then gently washed twice with PBS to remove cellular debris. Subsequently, the cells were treated with HA15 (20 μmol/L), ISRIB (10 μmol/L), and 4μ8C (15 μmol/L) in a medium containing 2% FBS (*n* = 6 per group). Immediately following the scratch (0 h), reference points were marked on the plate, and images were captured using an inverted microscope, ensuring the reference points remained visible. The plates were then returned to the incubator and maintained at 37°C with 5% CO_2 for 48 h. Additional images were taken at the same marked locations after 12, 24, 36, and 48 h. For data analysis, the wound area at each time point was measured using ImageJ (version 1.54g, National Institutes of Health). The percentage of wound closure was calculated with the following formula: Percentage of wound closure (x h) = [(Area at 0 h − Area at x h) / Area at 0 h] × 100.

Invasion assay

Matrigel (Corning, Cat. #354234) was first thawed at 4°C overnight. The following day, it was diluted with cold serum-free medium at a 1:2 ratio. The diluted Matrigel (100 μL) was added to each Transwell insert (8 μm; Corning, Cat. #3422) and incubated at 37°C for 2 h to form a gel layer. Cells were trypsinized, counted, and resuspended in serum-free medium at a density of 5×10^5 cells/mL. For the assay setup, 500 μL of complete medium containing serum as a chemoattractant was added to the lower chamber of a 24-well plate. Then, 200 μL of the cell suspension (containing 1×10^5 cells) was seeded into the upper chamber of each Matrigel-coated insert. HA15 (20 μmol/L), ISRIB (10 μmol/L), and 4μ8C (15 μmol/L) were applied to both the upper and lower chambers, where indicated (*n* = 6 per group). The plate was incubated at 37°C with 5% CO_2 for 48 h. After incubation, non-invaded cells on the upper surface of the membrane were carefully removed using a cotton swab. The invaded cells on the lower surface were fixed with 70% ethanol for 15 minutes and stained with Giemsa for another 15 minutes. The inserts were then rinsed three times with PBS and air-dried. Invaded cells were counted under a microscope in at least five random fields per insert. The mean number of invaded cells and the standard deviation were calculated for each condition.

Quantitative reverse transcription polymerase chain reaction (RT-qPCR)

The RT-qPCR method for gene expression analysis began with RNA extraction using the TaKaRa MiniBEST Universal RNA Extraction Kit (TaKaRa, Cat. #9767), where harvested cells were lysed, and RNA was purified through a series of binding, washing, and elution steps. The resulting RNA's concentration and purity (A260/A280 = ~2.0) were verified via spectrophotometry. Subsequently, 1 µg of this RNA was reverse transcribed into cDNA using the PrimeScript™ RT Master Mix (TaKaRa, Cat. #RR036A) in a 20 µL reaction, with incubation at 37°C for 15 minutes followed by enzyme inactivation at 85°C. For quantitative PCR, reactions were assembled in a 10 µL volume using TB Green® Premix Ex Taq™ (TaKaRa, Cat. #RR042A), with gene-specific primers (**Supplementary Table S4**). These reactions, prepared in triplicate, were amplified on a LightCycler 480 II (Roche, Cat. #5015243001) with a thermal profile of an initial denaturation at 95°C, followed by 40 cycles of denaturation (95°C) and annealing/extension (60°C), concluding with a melting curve analysis to confirm amplification specificity. Finally, the threshold cycle values were collected, and relative gene expression was calculated using the $2^{-\Delta\Delta Ct}$ method, normalizing target gene expression to an endogenous control (GAPDH) and comparing it to a calibrator sample, with rigorous quality controls applied throughout the process to ensure data reliability.

Luciferase labeling for *in vivo* imaging

Chondrosarcoma cells were harvested from three individual patients for each grade. Using a neon transfection kit (Invitrogen, Cat. #MPK1096), an aliquot of 1×10^6 cells was resuspended in 100 µL of resuspension buffer R along with 5 µg of the luciferase reporter transposon plasmid, pPB[Exp]-CAG>Luciferase (Vector Builder, VB900129-0843crf), and 2 µg of the transposase expression vector. Electroporation was performed using the Neon Transfection System (Invitrogen, NEON1S) with the following parameters: 1400 V, 20 ms pulse width, and 2 pulses. Immediately following electroporation, the cells were transferred to pre-warmed, antibiotic-free culture medium and incubated for 24 hours. To select for stable integrants, the medium was then replaced with a selection medium containing 1.0 µg/mL puromycin, which was maintained for 10 days with regular changes to remove non-transfected cells and allow for the expansion of resistant colonies. The resulting puromycin-resistant pools of cells were

expanded to establish stable luciferase-overexpressing cell lines. Luciferase expression was confirmed and quantified using the IVIS Spectrum imaging system (Revvity, Cat. #CLS148590) following the addition of a D-luciferin substrate (150 μg/mL; Sigma, Cat. #50227). Finally, the stable cell lines were characterized to ensure they retained parental cell characteristics, and frozen stocks were prepared for long-term storage, with regular monitoring to verify the stability of luciferase expression over multiple passages.

Subcutaneous patient-derived xenograft (PDX) experiment for tumor growth

For the subcutaneous tumor growth experiment, patient-derived tumor fragments (2-3 mm^3) were implanted into the right dorsum of anesthetized 8-week-old NSG mice. Following surgery, mice received standard post-operative analgesia and monitoring. Once the resulting tumors reached a volume of 100-150 mm^3, the mice were randomized into treatment groups (3 biological repeats per group). Treatments were administered intra-peritoneally once every 2 days and consisted of: vehicle control, HA15 (20 mg/kg), ISRIB (2.5 mg/kg), 4μ8C (20 mg/kg), and the combinations of ISRIB (2.5 mg/kg) + HA15 (20 mg/kg) and 4μ8C (20 mg/kg) + HA15 (20 mg/kg). Tumor dimensions were measured weekly using calipers, and the volume was calculated to generate growth curves for each group. Statistical analyses were performed to compare the efficacy of the different treatments in inhibiting tumor growth. All animal procedures were approved by the Committee on the Use of Live Animals in Teaching and Research of the University of Hong Kong (CULATR: 23-371).

Intraosseous luciferase experiment for metastasis and survival analysis

To model bone metastasis and assess survival, an intraosseous model was established by injecting a suspension of luciferase-expressing PDX cells (1×10^6 cells / 50 μL) directly into the femoral marrow cavity of anesthetized NSG mice. The mice (3 biological repeat per group) were randomized to receive the same treatment regimens as the subcutaneous study: vehicle control, HA15 (20 mg/kg), ISRIB (2.5 mg/kg), 4μ8C (20 mg/kg), ISRIB (2.5 mg/kg) + HA15 (20 mg/kg), and 4μ8C (20 mg/kg) + HA15 (20 mg/kg). Tumor progression and metastasis were monitored weekly via *in vivo* bioluminescence imaging. Mice were observed daily for 15 weeks, with survival as the primary endpoint. Clear humane endpoints, which included tumor

size >15 mm, weight loss >20%, signs of severe distress, inability to eat or drink, post-procedure complications, marked respiratory distress, temperature abnormalities (±3°C), or neurological deficits, with euthanasia performed using Pentobarbitone or Isoflurane, were predefined, and survival data were analyzed using Kaplan-Meier curves and Mantel-Cox tests to determine the impact of treatments on overall survival. All animal procedures were approved by the Committee on the Use of Live Animals in Teaching and Research of the University of Hong Kong (CULATR: 23-371).

Immunohistochemistry

Immunohistochemistry was performed on paraffin-embedded sections from a subcutaneous PDX tumor growth experiment. The sections were deparaffinized, rehydrated, and subjected to heat-induced epitope retrieval in citrate buffer (pH 6.0, 20 min). Endogenous peroxidase activity was blocked with 3% H_2O_2 for 10 min. The sections were then incubated overnight at 4°C with primary antibodies (**Supplementary Table S5**). This was followed by incubation with appropriate HRP-conjugated secondary antibodies for 1 h at room temperature. Signals were visualized using a DAB substrate, and hematoxylin was used for counterstaining. Negative controls (i.e., the omission of the primary antibody) were included in each staining batch to ensure specificity and assay validity. Quantification of immunohistochemical staining was performed using ImageJ (version 1.54g, National Institutes of Health). The expression level was quantified by measuring the integrated optical density (IOD) per unit area, which accounts for both the proportion of positive cells and the staining intensity.

Statistical analysis

Statistical analyses were performed using GraphPad Prism (version 8, Dotmatics) and SPSS (version 26, IBM), with significance levels indicated as * $P < 0.05$, ** $P < 0.01$, and *** $P < 0.001$. Normality was assessed using the Shapiro-Wilk test. For comparisons between two groups, an unpaired two-tailed *t*-test (normal distribution) or Mann-Whitney U test (non-normal distribution) was used; for multiple groups, one-way ANOVA with Tukey's post-hoc test or the Kruskal-Wallis test with Dunn's test was applied. Specific analyses included two-

way ANOVA for longitudinal wound healing data, non-linear regression for IC_{50} calculation, a mixed-effects model for *in vivo* tumor growth, and the log-rank test for Kaplan-Meier survival curves.

Supplementary Table S1. Patient cohort and tissue sample information

ID	Gender	Age	Type	Anatomical Site
LCHS1	Female	58 years	Low grade chondrosarcoma	Humerus
LCHS2	Male	35 years	Low grade chondrosarcoma	Pelvis
LCHS3	Male	51 years	Low grade chondrosarcoma	Femur
HCHS1	Female	69 years	High grade chondrosarcoma	Pelvis
HCHS2	Male	63 years	High grade chondrosarcoma	Pelvis
HCHS3	Female	68 years	High grade chondrosarcoma	Femur
NC1	Female	14 weeks (fetus)	Normal fetal chondrocytes	Epiphyseal region of fetal femur
NC2	Female	15 weeks (fetus)	Normal fetal chondrocytes	Epiphyseal region of fetal femur
NC3	Female	14 weeks (fetus)	Normal fetal chondrocytes	Epiphyseal region of fetal femur

Abbreviations: HCHS, high-grade chondrosarcoma; LCHS, low-grade chondrosarcoma; NC, normal chondrocytes.

Supplementary Table S2. ER stress modulating compounds

Name	Target	Brand	CAS No.	Cat. No.
HA15	HSPA5	MedChemExpress	1609402-14-3	HY-100437
ISRIB	eIF2α	MedChemExpress	1597403-47-8	HY-12495
4μ8C	IRE1α	MedChemExpress	14003-96-4	HY-19707

Abbreviations: CAS, Chemical Abstracts Service; eIF2α, eukaryotic translation initiation factor 2 alpha; HSPA5, heat shock protein family A member 5; IRE1α, inositol-requiring enzyme 1 alpha.

Supplementary Table S3. Small interfering RNA (siRNA)

Name	Guide Sequence (5' ➔ 3')	Passenger (5' ➔ 3')
siATF5	UCUAAAAUAUUUCCUUUUGGC	CAAAAGGAAAUAUUUUAGAGG
siDDIT3	UGUUCUUUCUCCUUCAUGCGC	GCAUGAAGGAGAAAGAACAGG

Abbreviations: ATF5, activating transcription factor 5; DDIT3, DNA damage-inducible transcript 3.

Supplementary Table S4. Primers for quantitative PCR

Name	Forward Sequence (5' ➔ 3')	Reverse Sequence (5' ➔ 3')
HSPA5	CATCACGCCGTCCTATGTCG	CGTCAAAGACCGTGTTCTCG
PERK	ACGATGAGACAGAGTTGCGAC	ATCCAAGGCAGCAATTCTCCC
EIF2S1	TGGTGAATGTCAGATCCATTGC	TAGAACGGATACGCCTTCTGG
ATF4	ATGACCGAAATGAGCTTCCTG	GCTGGAGAACCCATGAGGT
ATF5	TGGCTCGTAGACTATGGGAAA	ATCAACTCGCTCAGTCATCCA
DDIT3	GGAAACAGAGTGGTCATTCCC	CTGCTTGAGCCGTTCATTCTC
ATF6	TCCTCGGTCAGTGGACTCTTA	CTTGGGCTGAATTGAAGGTTTTG
ERN1	CACAGTGACGCTTCCTGAAAC	GCCATCATTAGGATCTGGGAGA
TRAF2	TCCCTGGAGTTGCTACAGC	AGGCGGAGCACAGGTACTT
ASK1	CTGCATTTTGGGAAACTCGACT	AAGGTGGTAAAACAAGGACGG
MKK7	CCACGTCATTGCCGTTAAGC	GCACGATGTAGGGGCAGTC
JNK	TGTGTGGAATCAAGCACCTTC	AGGCGTCATCATAAAACTCGTTC
JUN	TCCAAGTGCCGAAAAAGGAAG	CGAGTTCTGAGCTTTCAAGGT
FOS	CCGGGGATAGCCTCTCTTACT	CCAGGTCCGTGCAGAAGTC
XBP1	CCCTCCAGAACATCTCCCCAT	ACATGACTGGGTCCAAGTTGT
NFKB1	AACAGAGAGGATTTCGTTTCCG	TTTGACCTGAGGGTAAGACTTCT
RELA	ATGTGGAGATCATTGAGCAGC	CCTGGTCCTGTGTAGCCATT
BCL2	GGTGGGGTCATGTGTGTGG	CGGTTCAGGTACTCAGTCATCC
NOXA	ACCAAGCCGGATTTGCGATT	ACTTGCACTTGTTCCTCGTGG
BAX	CCCGAGAGGTCTTTTTCCGAG	CCAGCCCATGATGGTTCTGAT
DR5	ATGGAACAACGGGGACAGAAC	CTGCTGGGGAGCTAGGTCT
CASP8	TTTCTGCCTACAGGGTCATGC	GCTGCTTCTCTCTTTGCTGAA
CASP3	CATGGAAGCGAATCAATGGACT	CTGTACCAGACCGAGATGTCA
PARP1	CGGAGTCTTCGGATAAGCTCT	TTTCCATCAAACATGGGCGAC
MMP2	TACAGGATCATTGGCTACACACC	GGTCACATCGCTCCAGACT
MMP9	TGTACCGCTATGGTTACACTCG	GGCAGGGACAGTTGCTTCT
VIM	GACGCCATCAACACCGAGTT	CTTTGTCGTTGGTTAGCTGGT

CCND1	GCTGCGAAGTGGAAACCATC	CCTCCTTCTGCACACATTTGAA
CDK4	ATGGCTACCTCTCGATATGAGC	CATTGGGGACTCTCACACTCT
CDK6	GCTGACCAGCAGTACGAATG	GCACACATCAAACAACCTGACC
E2F1	ACGCTATGAGACCTCACTGAA	TCCTGGGTCAACCCCTCAAG
KI67	ACGCCTGGTTACTATCAAAAGG	CAGACCCATTTACTTGTGTTGGA
PCNA	CCTGCTGGGATATTAGCTCCA	CAGCGGTAGGTGTCGAAGC
IGFBP7	CGAGCAAGGTCCTTCCATAGT	GGTGTCGGGATTCCGATGAC
TIMP2	AAGCGGTCAGTGAGAAGGAAG	GGGGCCGTGTAGATAAACTCTAT
GAPDH	GGAGCGAGATCCCTCCAAAAT	GGCTGTTGTCATACTTCTCATGG

Abbreviations: ASK1, apoptosis signal-regulating kinase 1; ATF4, activating transcription factor 4; ATF5, activating transcription factor 5; ATF6, activating transcription factor 6; BAX, BCL2 associated X; BCL2, B-cell lymphoma 2; CASP3, caspase 3; CASP8, caspase 8; CCND1, G1/S-specific cyclin D1; CDK4, cyclin-dependent kinase 4; CDK6, cyclin-dependent kinase 6; DDIT3, DNA damage-inducible transcript 3; DR5, death receptor 5; E2F1, E2F transcription factor 1; EIF2S1, eukaryotic translation initiation factor 1A; ERN1, endoplasmic reticulum to nucleus signaling 1 (known as inositol-requiring enzyme 1); FOS, fos proto-oncogene; GAPDH, glyceraldehyde-3-phosphate dehydrogenase; HSPA5, heat shock protein family A member 5; IGFBP7, insulin like growth factor binding protein 7; JNK, c-Jun N-terminal kinase; JUN, jun proto-oncogene; KI67, marker of proliferation Ki-67; MKK7, mitogen-activated protein kinase kinase 7; MMP2, matrix metallopeptidase 2; MMP9, matrix metallopeptidase 9; NFKB1, nuclear factor kappa B subunit 1; NOXA, phorbol-12-myristate-13-acetate-induced protein 1; PARP1, poly [ADP-ribose] polymerase 1; PCNA, proliferating cell nuclear antigen; PERK, protein kinase R-like ER kinase; RELA, rela proto-oncogene; TIMP2, tissue inhibitor of metalloproteinase 2; TRAF2, TNF receptor-associated factor 2; VIM, vimentin; XBP1, X-box binding protein 1.

Supplementary Table S5. Antibodies for immunohistochemistry

Name	Reacts with	Host species	Brand	Cat. No.	Dilution
anti-HSPA5	Human	Rabbit	Abcam	ab21685	1:2,000
anti-ATF5	Human	Rabbit	Abcam	ab184923	1:2,000
anti-DDIT3	Human	Mouse	Abcam	ab11419	1:2,000
anti-c-CASP3	Human	Rabbit	Cell Signaling Technology	#9664	1:2,000
anti-Ki-67	Human	Rabbit	Abcam	ab15580	1:2,000
anti-MMP9	Human	Rabbit	Abcam	ab283575	1:2,000
anti-Rabbit IgG	Rabbit	Goat	Invitrogen	31460	1:5,000
anti-Mouse IgG	Mouse	Goat	Invitrogen	31430	1:5,000

Abbreviations: ATF5, activating transcription factor 5; c-CASP3, cleaved caspase 3; DDIT3, DNA damage-inducible transcript 3; HSPA5, heat shock protein family A member 5; Ki-67, marker of proliferation Ki-67; MMP9, matrix metallopeptidase 9.

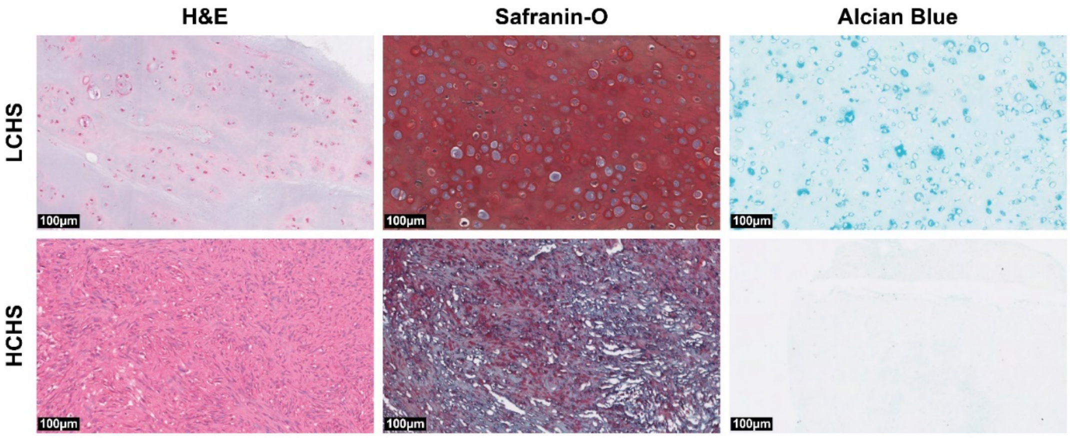

Supplementary Figure S1. Histopathological comparison of low-grade (upper row) and high-grade (lower row) chondrosarcoma (scale: 100 μm). H&E: LCHS has dispersed cells in a pale matrix; HCHS has a dense fibrous matrix with scattered nuclei. Safranin-O: LCHS shows defined purple/blue structures on red background; HCHS displays complex purple-red/blue fibrous patterns. Alcian blue: Distinct blue structures in LCHS; minimal/no blue staining (white appearance) in HCHS. Diagnoses confirmed by a musculoskeletal pathologist. Abbreviations: H&E, hematoxylin and eosin; HCHS, high-grade chondrosarcoma; LCHS, low-grade chondrosarcoma.

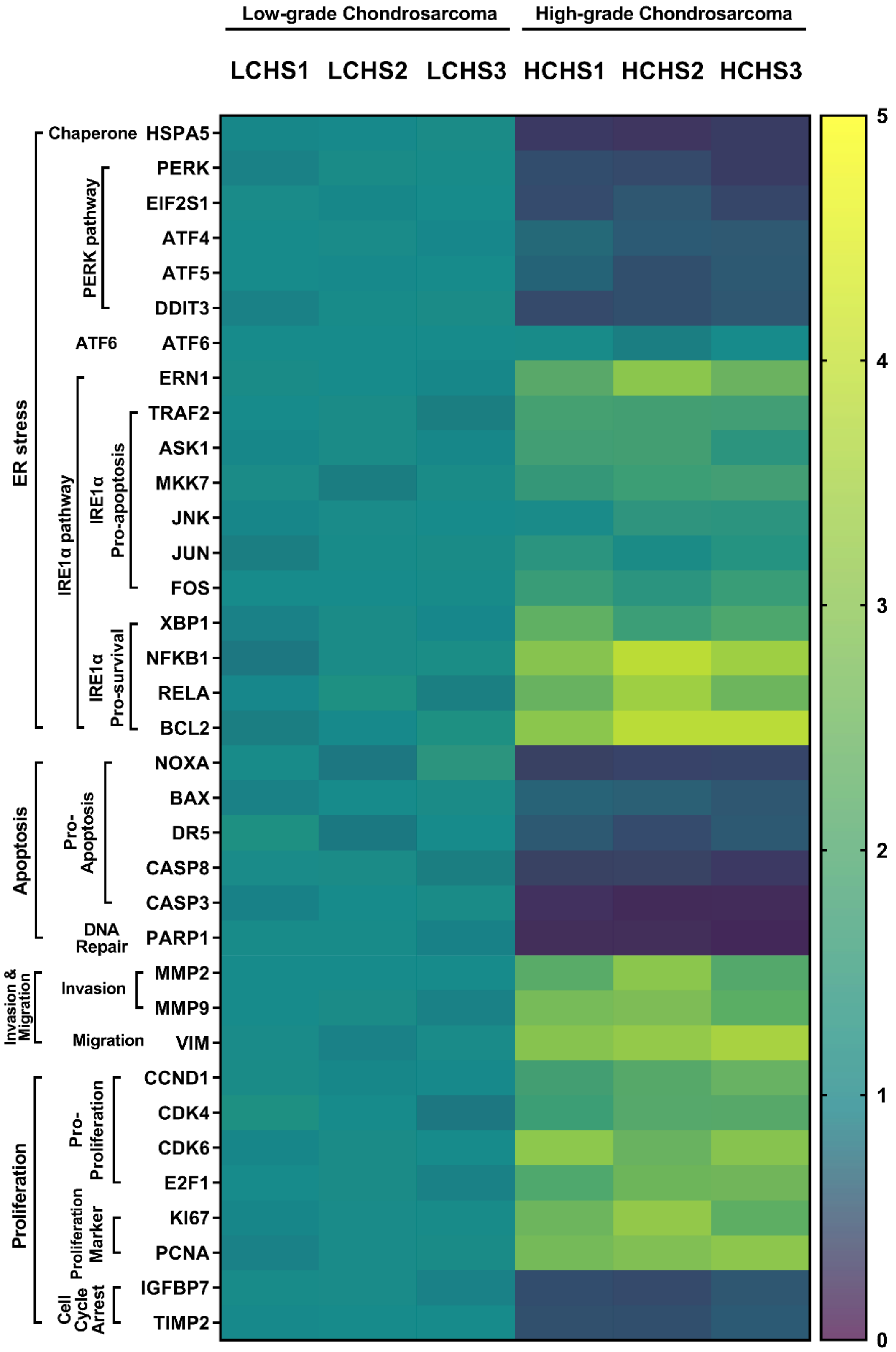

Supplementary Figure S2. Baseline gene expression comparison between LCHS and HCHS by RT-qPCR (purple to yellow: 0 to 5 fold change). LCHS is characterized by a highly activated PERK pathway (PERK, EIF2S1, ATF4, ATF5, and DDIT3), with elevated expression of apoptotic markers (NOXA, BAX, DR5, CASP8, and CASP3) and cell cycle arrest markers (IGFBP7 and TIMP2). In

contrast, HCHS shows activation of the IRE1α pro-survival pathway (ERN1, XBP1, NFKB1, RELA, and BCL2) and increased expression of markers associated with proliferation (CCND1, CDK4, CDK6, E2F1, KI67 and PCNA) and migration (MMP2, MMP9 and VIM). Each group consisted of three independent samples (biological replicates), and each sample was analyzed in three technical replicates. Abbreviations: ASK1, apoptosis signal-regulating kinase 1; ATF4, activating transcription factor 4; ATF5, activating transcription factor 5; ATF6, activating transcription factor 6; BAX, BCL2 associated X; BCL2, B-cell lymphoma 2; CASP3, caspase 3; CASP8, caspase 8; CCND1, G1/S-specific cyclin D1; CDK4, cyclin-dependent kinase 4; CDK6, cyclin-dependent kinase 6; DDIT3, DNA damage-inducible transcript 3; DR5, death receptor 5; E2F1, E2F transcription factor 1; EIF2S1, eukaryotic translation initiation factor 2 subunit alpha (eIF2α); ERN1, endoplasmic reticulum to nucleus signaling 1 (IRE1α); FOS, fos proto-oncogene; HCHS, high-grade chondrosarcoma; HSPA5, heat shock protein family A member 5; IGFBP7, insulin like growth factor binding protein 7; JNK, c-Jun N-terminal kinase; JUN, jun proto-oncogene; KI67, marker of proliferation Ki-67 antigen; LCHS, low-grade chondrosarcoma; MKK7, mitogen-activated protein kinase kinase 7; MMP2, matrix metallopeptidase 2; MMP9, matrix metallopeptidase 9; NFKB1, nuclear factor kappa B subunit 1; NOXA, phorbol-12-myristate-13-acetate-induced protein 1; PARP1, poly [ADP-ribose] polymerase 1; PCNA, proliferating cell nuclear antigen; PERK, protein kinase R-like endoplasmic reticulum kinase; RELA, rela proto-oncogene; TIMP2, tissue inhibitor of metalloproteinase 2; TRAF2, TNF receptor-associated factor 2; VIM, vimentin; XBP1, X-box binding protein 1.

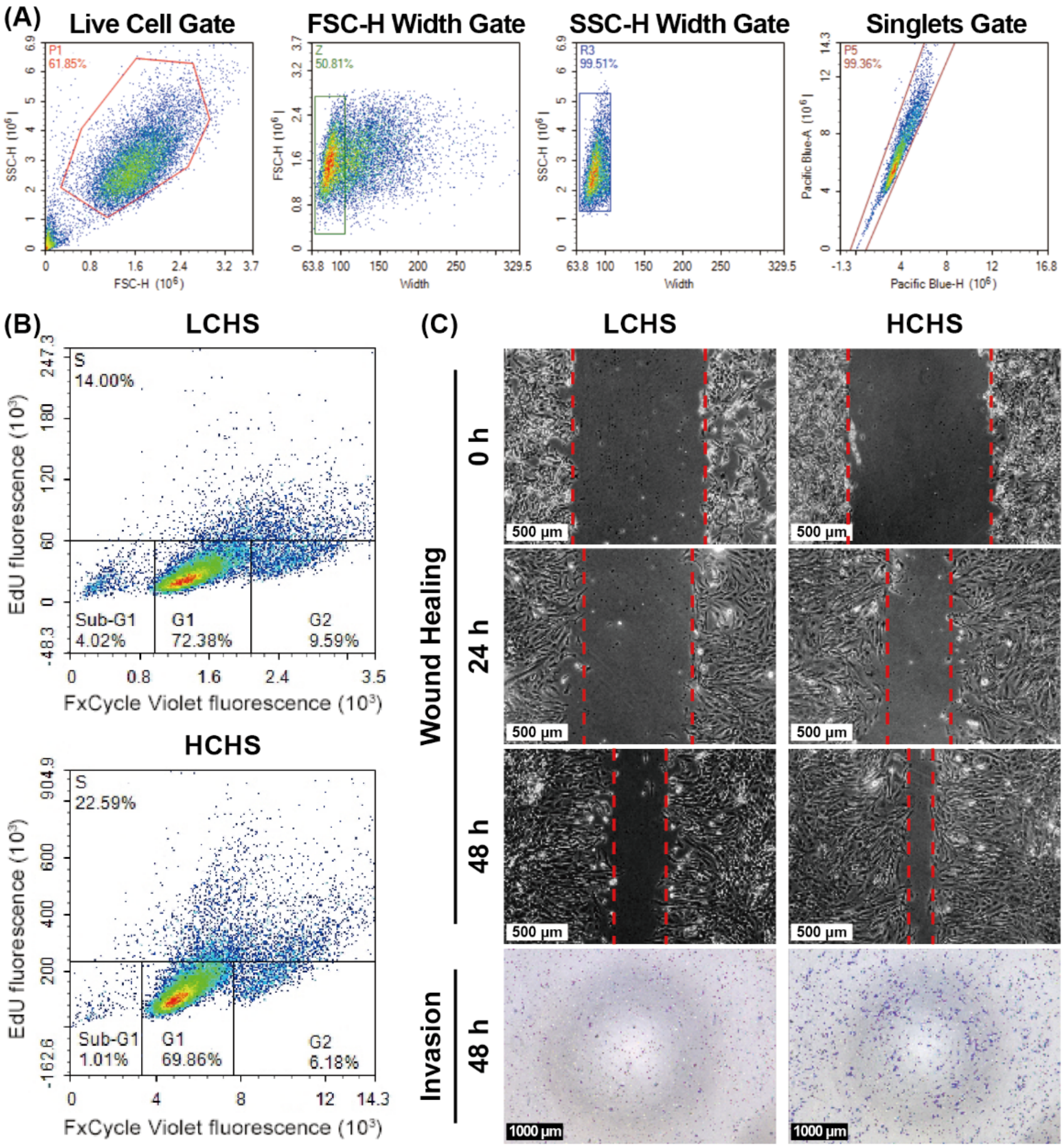

Supplementary Figure S3. (A) The gating strategy of cell cycle analysis using the Click-iT Plus EdU Alexa Fluor 488 Flow Cytometry Assay Kit and FxCycle Violet Stain, sequentially excluded debris and selected single cells. **(B)** Representative plots of cell cycle analysis for LCHS and HCHS cells (*n* = 3 per group). **(C)** Representative images of scratch wound healing assays (0 h, 12 h, 48 h; scale: 500 µm) and Transwell invasion assay (48 h; scale: 1,000 µm) for LCHS and HCHS cells (*n* = 6 per group). Abbreviations: HCHS, high-grade chondrosarcoma; LCHS, low-grade chondrosarcoma.

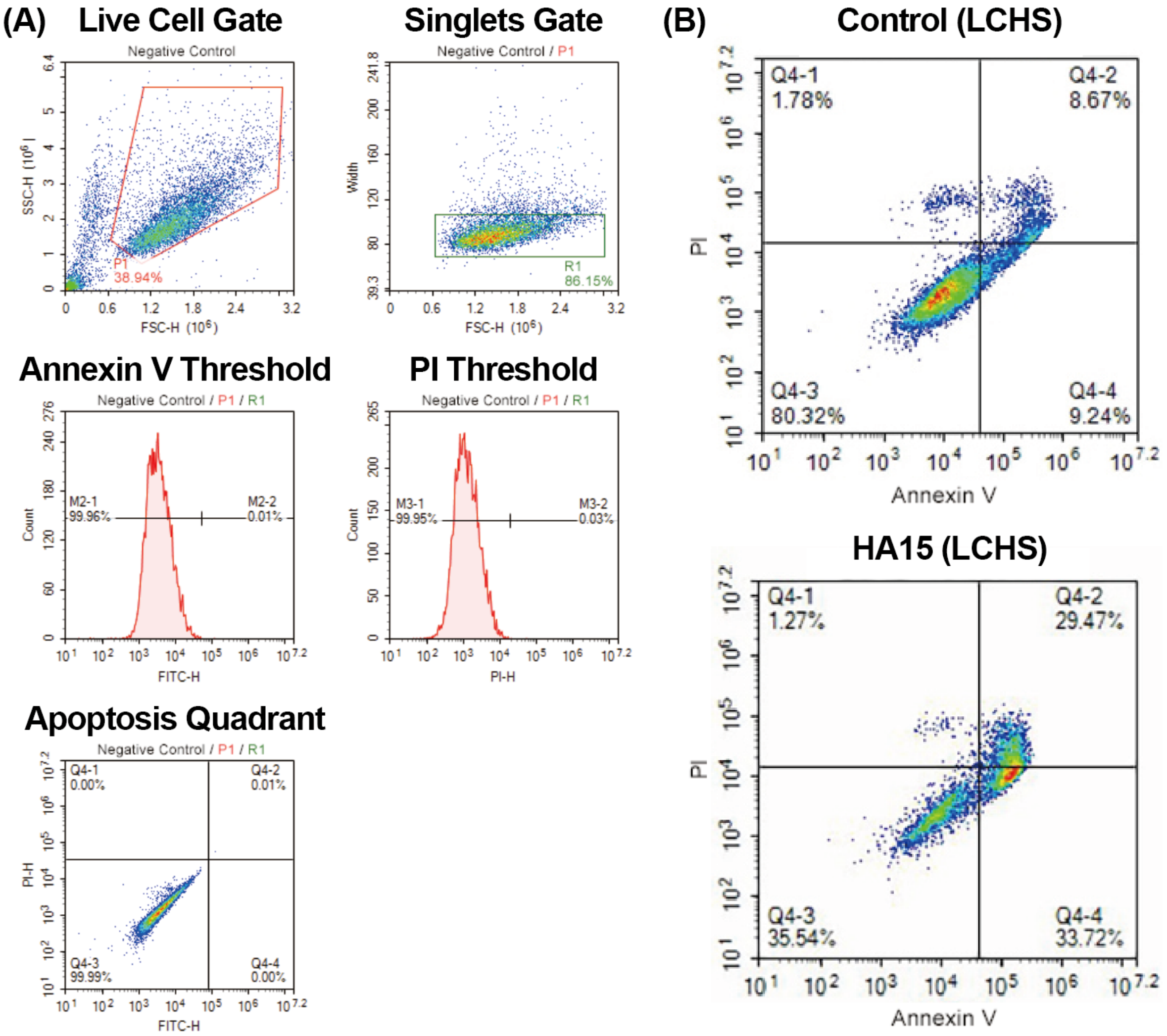

Supplementary Figure S4. (A) The gating strategy for Annexin V/PI apoptosis analysis sequentially excluded debris, selected single cells, and applied fluorescence thresholds defined by the negative control to delineate live, early apoptotic, and late apoptotic populations in the final quadrant plot; **(B)** Representative plots of Annexin V/PI apoptosis analysis for LCHS cells without or with treatment of HA15 (20 µmol/L; *n* = 3 per group). Abbreviations: LCHS, low-grade chondrosarcoma.

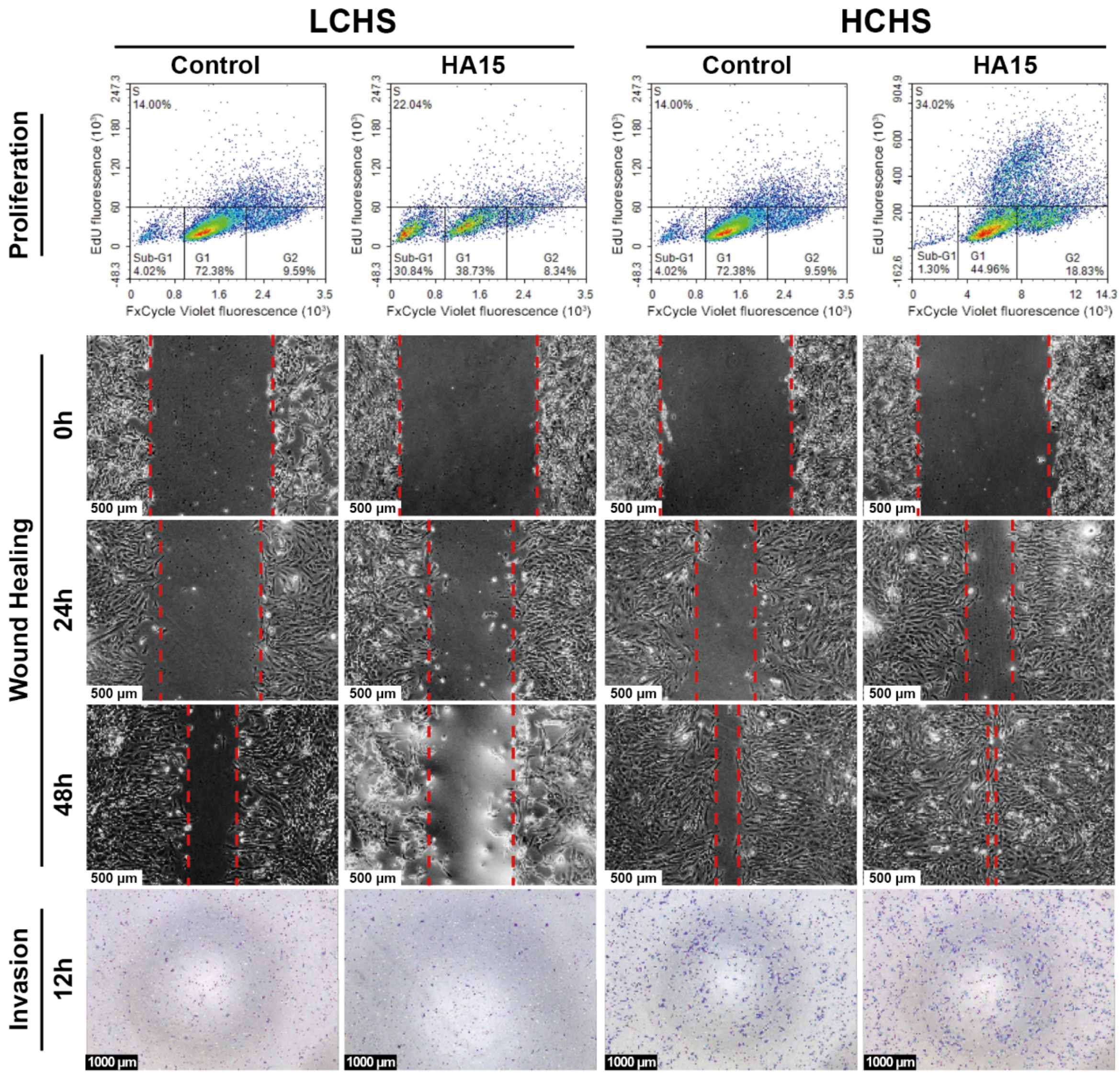

Supplementary Figure S5. Representative images of cell cycle assay, scratch wound healing assays (0 h, 12 h, 48 h; scale 500 μm) and Transwell invasion assay (48 h; scale 1,000 μm) for LCHS and HCHS cells under control and HA15 monotherapy (20 μmol/L; *n* = 6 per group). The gating strategy is shown in Supplementary Figure S3A. Abbreviations: HCHS, high-grade chondrosarcoma; LCHS, low-grade chondrosarcoma.

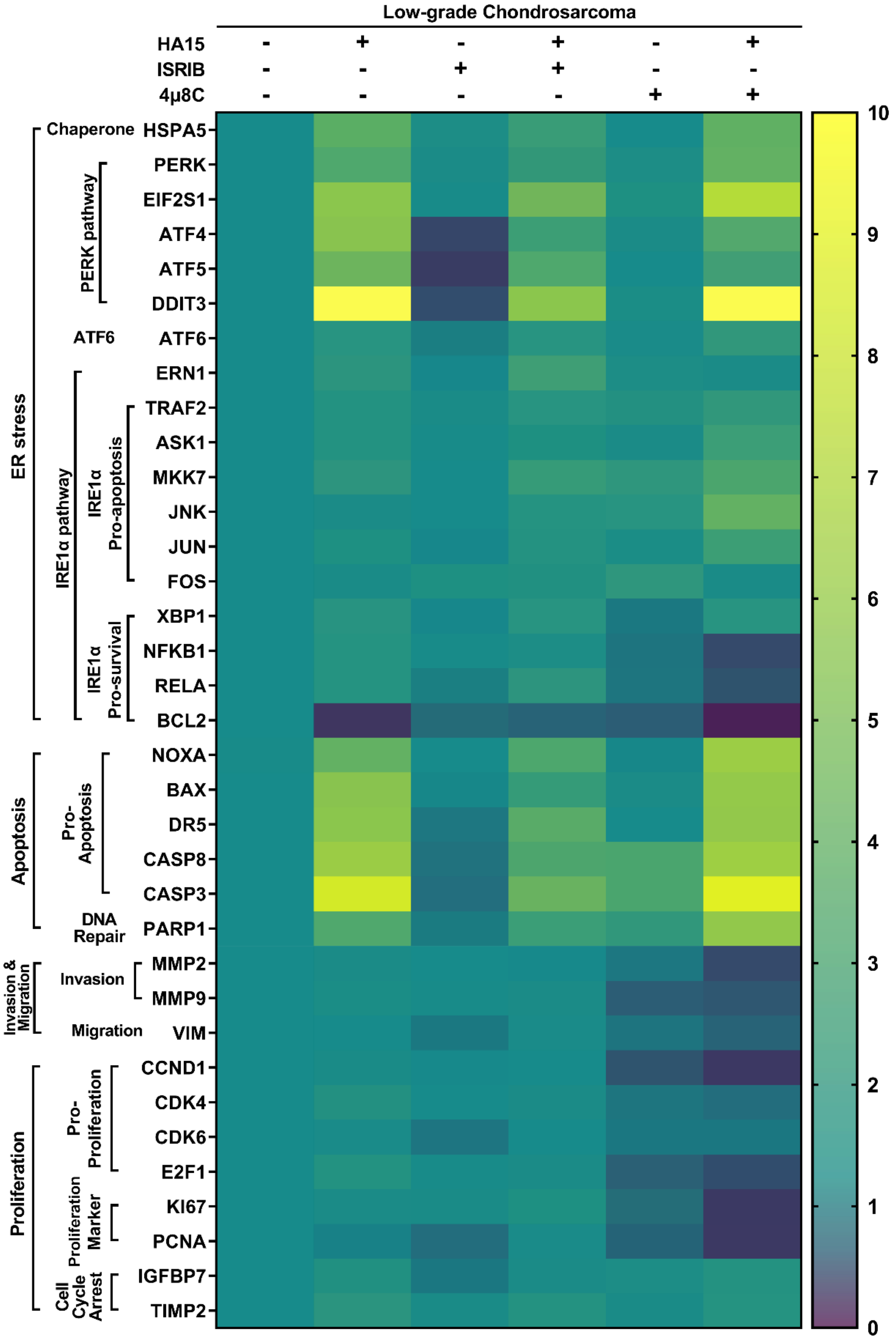

Supplementary Figure S6. Differential gene expression in LCHS cells treated with HA15 (20 μmol/L), ISRIB (10 μmol/L), and 4μ8C (15 μmol/L) by RT-qPCR (purple to yellow: 0 to 10 fold change). HA15 induced apoptosis primarily by activating the PERK pathway. This led to increased levels of

downstream expressions (PERK, EIF2S1, ATF4, ATF5, DDIT3) and ultimately elevated expression of pro-apoptotic factors like NOXA, BAX, DR5, CASP8, CASP3, and PARP1, compared to the control group. ISRIB inhibited the apoptotic effects of HA15 ($n = 3$ per group). Abbreviations: ASK1, apoptosis signal-regulating kinase 1; ATF4, activating transcription factor 4; ATF5, activating transcription factor 5; ATF6, activating transcription factor 6; BAX, BCL2 associated X; BCL2, B-cell lymphoma 2; CASP3, caspase 3; CASP8, caspase 8; CCND1, G1/S-specific cyclin D1; CDK4, cyclin-dependent kinase 4; CDK6, cyclin-dependent kinase 6; DDIT3, DNA damage-inducible transcript 3; DR5, death receptor 5; E2F1, E2F transcription factor 1; EIF2S1, eukaryotic translation initiation factor 2 subunit alpha (eIF2α); ERN1, endoplasmic reticulum to nucleus signaling 1 (IRE1α); FOS, fos proto-oncogene; HSPA5, heat shock protein family A member 5; IGFBP7, insulin like growth factor binding protein 7; JNK, c-Jun N-terminal kinase; JUN, jun proto-oncogene; KI67, marker of proliferation Ki-67; MKK7, mitogen-activated protein kinase kinase 7; MMP2, matrix metallopeptidase 2; MMP9, matrix metallopeptidase 9; NFKB1, nuclear factor kappa B subunit 1; NOXA, phorbol-12-myristate-13-acetate-induced protein 1; PARP1, poly [ADP-ribose] polymerase 1; PCNA, proliferating cell nuclear antigen; PERK, protein kinase R-like ER kinase; RELA, rela proto-oncogene; TIMP2, tissue inhibitor of metalloproteinase 2; TRAF2, TNF receptor-associated factor 2; VIM, vimentin; XBP1, X-box binding protein 1.

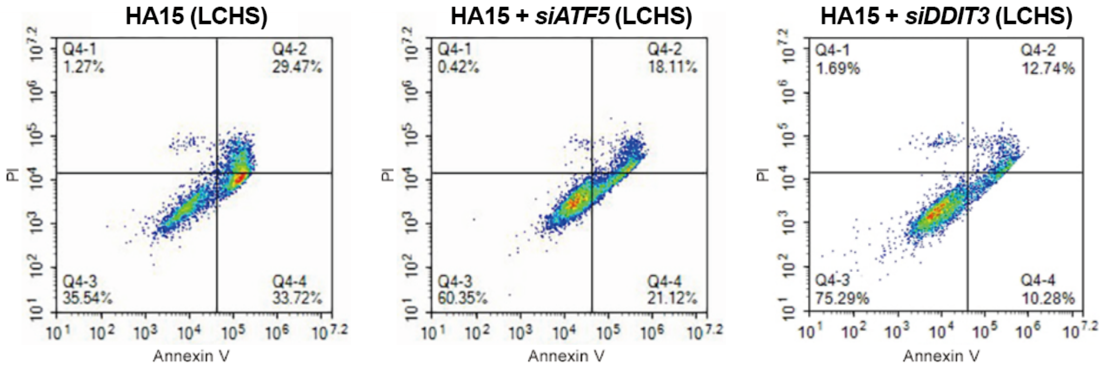

Supplementary Figure S7. Representative plots of Annexin V/PI apoptosis analysis for LCHS cells treated with 20 μmol/L HA15 alone, HA15 + *siATF5* knockdown, or HA15 + *siDDIT3* knockdown ($n = 3$ per group). The gating strategy is shown in Supplementary Figure S4A. Abbreviations: LCHS, low-grade chondrosarcoma; ATF5, activating transcription factor 5; DDIT3, DNA damage inducible transcript 3.

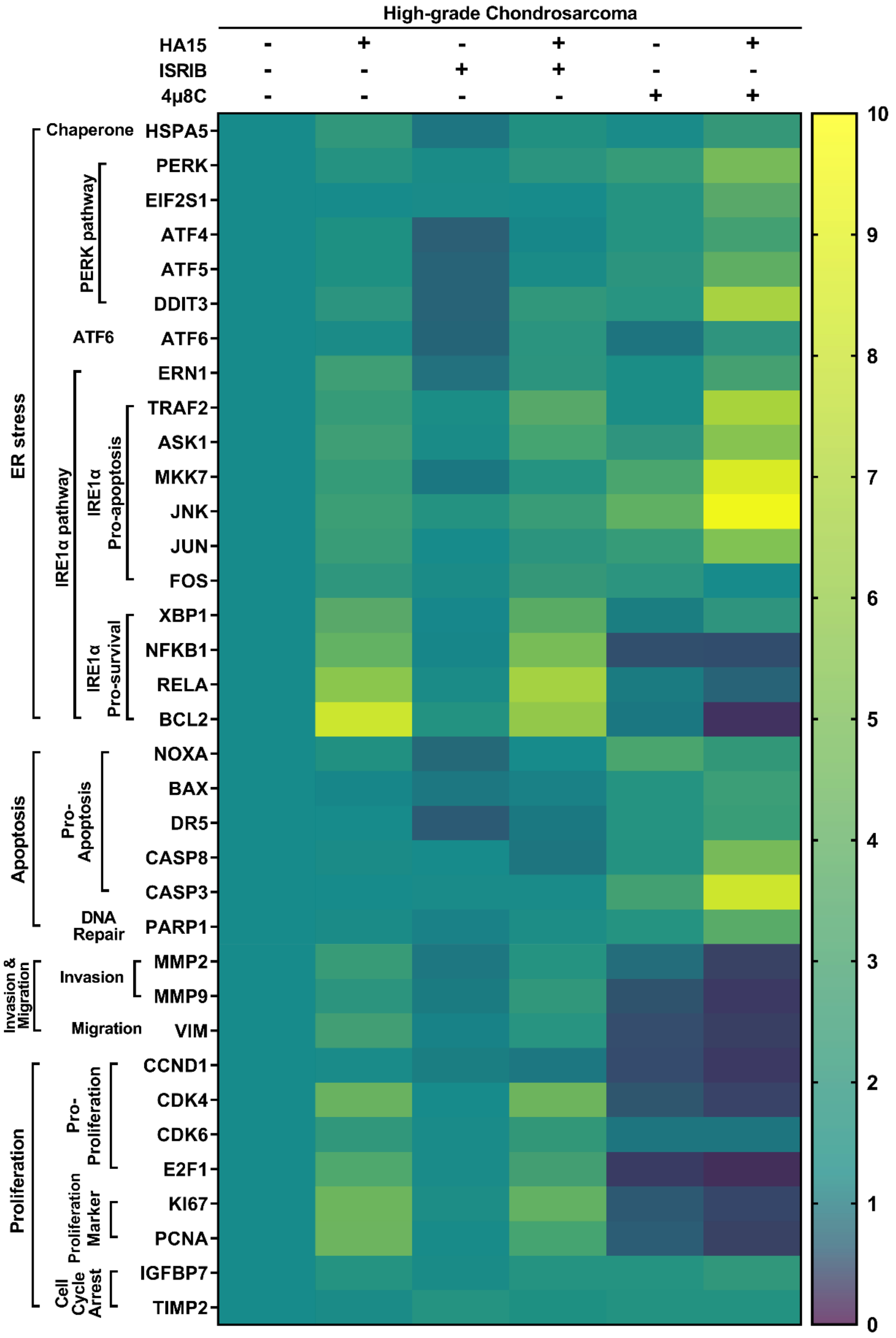

Supplementary Figure S8. Differential gene expression analysis in HCHS treated with HA15 (20 μmol/L), ISRIB (10 μmol/L), and 4μ8C (15 μmol/L) by RT-qPCR (purple to yellow: 0 to 10 fold change). HA15 preferentially activates the IRE1α pro-survival pathway (ERN1, XBP1, NFKB1, RELA and BCL2). This activation was associated with the upregulation of

proliferation-related markers (CCND1, CDK4, CDK6, E2F1, KI67, and PCNA) and migration/invasion related markers (MMP2, MMP9, and VIM) in HCHS. 4μ8C redirects HA15-induced ER stress signals toward the pro-apoptotic PERK/p-eIF2α/DDIT3 and IRE1α/TRAF2/JNK pathways (TRAF2, ASK1, MKK7, JNK, JUN), suppressing proliferation- and migration-related markers (*n* = 3 per group). Abbreviations: ASK1, apoptosis signal-regulating kinase 1; ATF4, activating transcription factor 4; ATF5, activating transcription factor 5; ATF6, activating transcription factor 6; BAX, BCL2 associated X; BCL2, B-cell lymphoma 2; CASP3, caspase 3; CASP8, caspase 8; CCND1, G1/S-specific cyclin D1; CDK4, cyclin-dependent kinase 4; CDK6, cyclin-dependent kinase 6; DDIT3, DNA damage-inducible transcript 3; DR5, death receptor 5; E2F1, E2F transcription factor 1; EIF2S1, eukaryotic translation initiation factor 2 subunit alpha (eIF2α); ERN1, endoplasmic reticulum to nucleus signaling 1 (IRE1α); FOS, fos proto-oncogene; HSPA5, heat shock protein family A member 5; IGFBP7, insulin like growth factor binding protein 7; JNK, c-Jun N-terminal kinase; JUN, jun proto-oncogene; KI67, marker of proliferation Ki-67; MKK7, mitogen-activated protein kinase kinase 7; MMP2, matrix metallopeptidase 2; MMP9, matrix metallopeptidase 9; NFKB1, nuclear factor kappa B subunit 1; NOXA, phorbol-12-myristate-13-acetate-induced protein 1; PARP1, poly [ADP-ribose] polymerase 1; PCNA, proliferating cell nuclear antigen; PERK, protein kinase R-like ER kinase; RELA, rela proto-oncogene; TIMP2, tissue inhibitor of metalloproteinase 2; TRAF2, TNF receptor-associated factor 2; VIM, vimentin; XBP1, X-box binding protein 1.

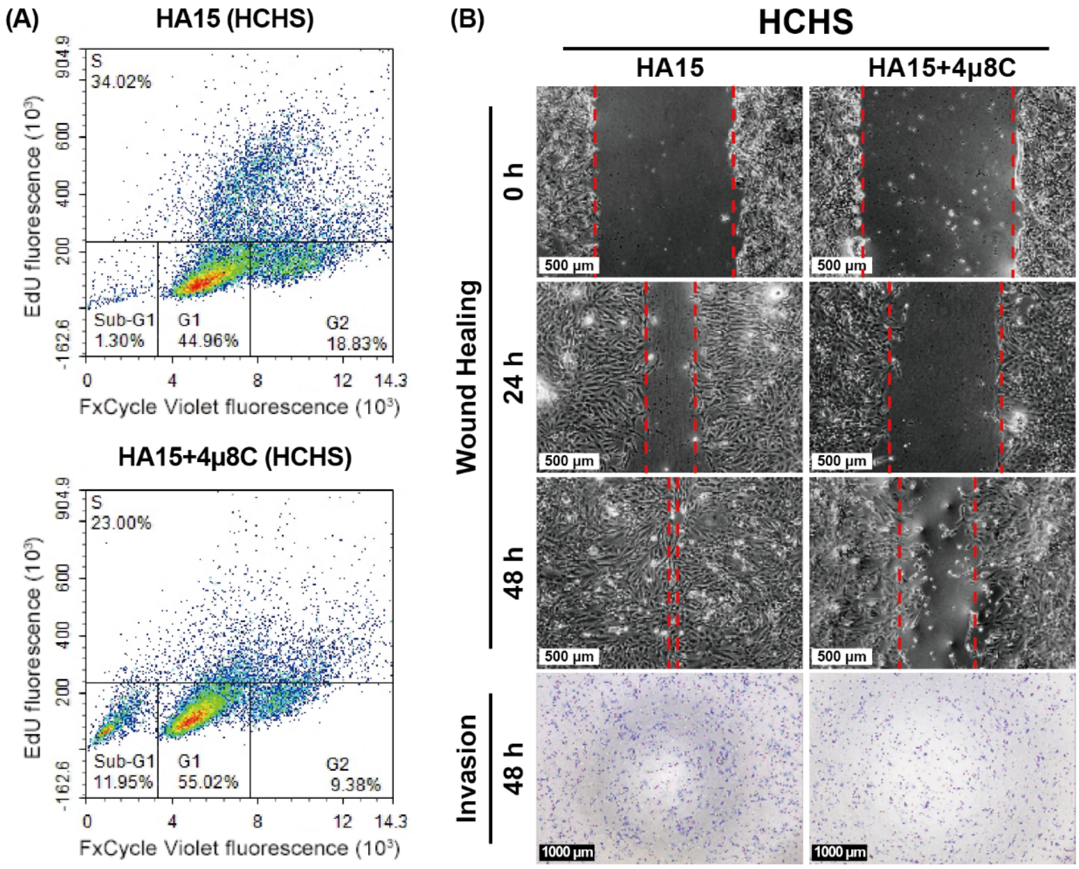

Supplementary Figure S9. (A) Representative plots of cell cycle analysis for HCHS cells treated with HA15 (20 μmol/L) and HA15 + 4μ8C (20 μmol/L + 15 μmol/L, respectively) (*n* = 3 per group). **(B)** Representative images of scratch wound healing assays (0 h, 12 h, 48 h; scale: 500 μm) and Transwell invasion assay (48 h; scale: 1,000 μm) in HCHS cells under HA15 monotherapy (20 μmol/L) and HA15 + 4μ8C combination therapy (20 μmol/L + 15 μmol/L, respectively) (*n* = 6 per group). The gating strategy is shown in Supplementary Figure S3A. Abbreviations: HCHS, high-grade chondrosarcoma.

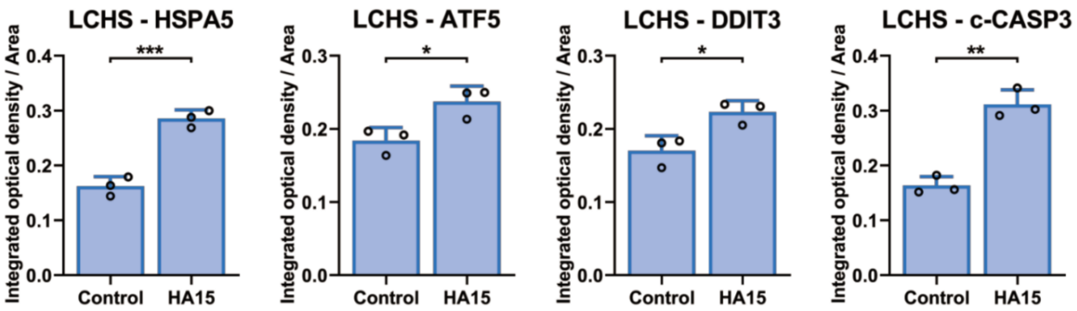

Supplementary Figure S10. Immunohistochemical quantification of ER stress and apoptosis markers in LCHS harvested from subcutaneous PDX models. Quantified expression levels measured as integrated optical density per area. Compared to control, HA15 (20 mg/kg, i.p., qod) increased the immunoreactivity of HSPA5 (0.16 vs. 0.29), ATF5 (0.18 vs. 0.24), DDIT3 (0.17 vs. 0.22), and c-CASP3 (0.16 vs. 0.31). Statistical significance is denoted by asterisks (* $P < 0.05$, ** $P < 0.01$, *** $P < 0.001$; unpaired *t*-test). Abbreviations: ATF5, activating transcription factor 5; c-CASP3, cleaved caspase 3; DDIT3, DNA damage-inducible transcript 3; HSPA5, heat shock protein family A member 5; i.p., intraperitoneally; LCHS, low-grade chondrosarcoma; qod, every other day.

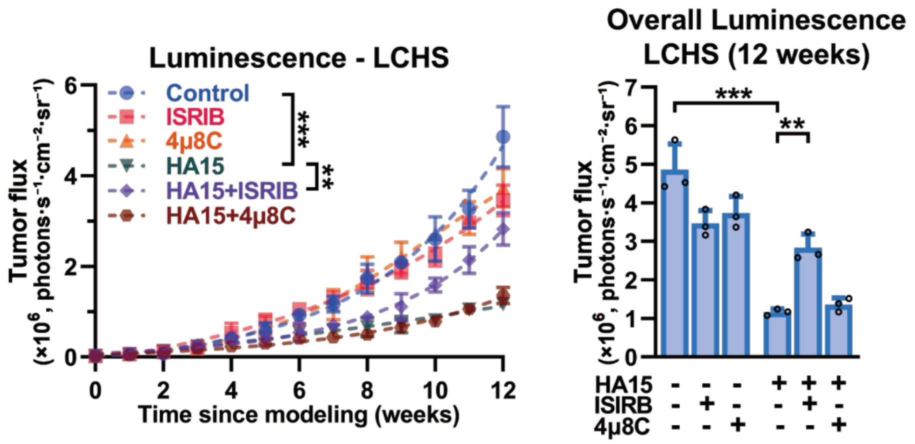

Supplementary Figure S11. Real-time bioluminescence and quantification of LCHS tumor burden over a 12-week treatment period. The left panel shows real-time bioluminescence of LCHS tumor burden over 12 weeks, while the right panel displays the endpoint quantification, both comparing treatments of control, HA15 (20 mg/kg, i.p., qod), ISRIB (2.5 mg/kg, i.p., qod), and HA15+ISRIB (20 mg/kg + 2.5 mg/kg, i.p., qod, respectively). Quantification of tumor flux is presented as photons·s^{-1}·cm^{-2}·sr^{-1} (×10^6). Compared to control, treatment with HA15 alone resulted in a significant, approximately 4.2-fold reduction in bioluminescent signal (from 4.86 to 1.16). Compared to HA15 monotherapy, combined treatment with HA15 and ISRIB led to a significant, approximately 2.4-fold increase in bioluminescent signal (from 1.16 to 2.83). Statistical significance is denoted by asterisks (* $P < 0.05$, ** $P < 0.01$, *** $P < 0.001$; one-way ANOVA test). Abbreviations: i.p., intraperitoneally; LCHS, low-grade chondrosarcoma; qod, every other day.

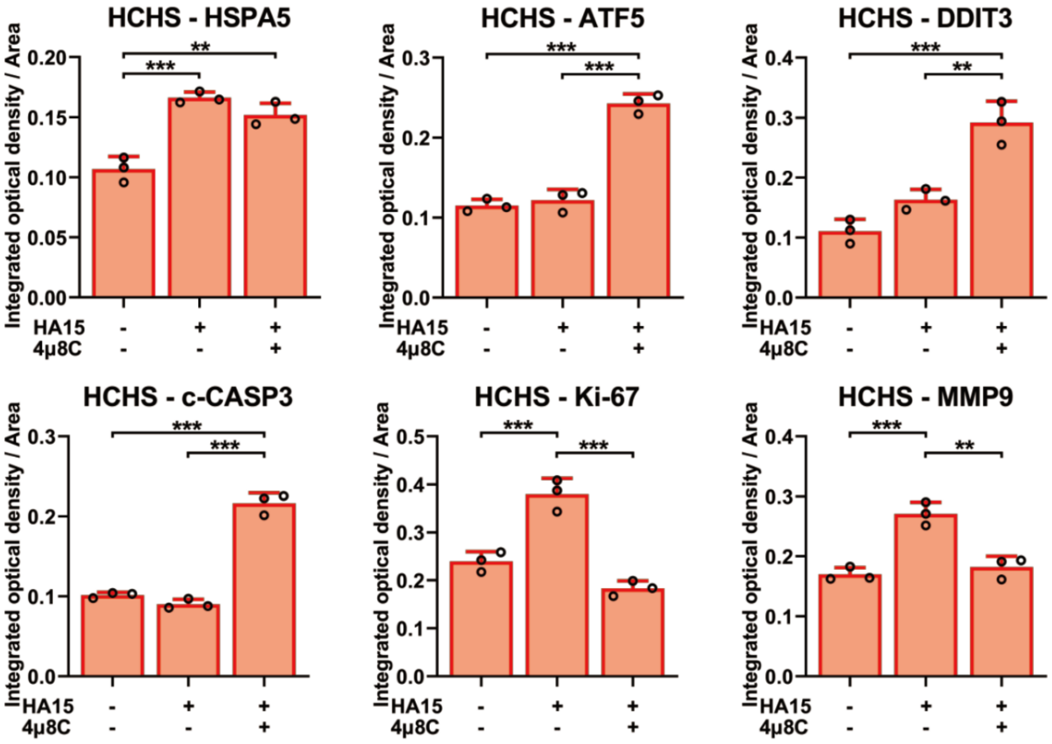

Supplementary Figure S12. Immunohistochemical quantification of ER stress, apoptosis, proliferation, and migration markers in HCHS harvested from subcutaneous PDX models with treatments of control, HA15 (20 mg/kg, i.p., qod), and HA15 + 4μ8C (20 mg/kg + 20 mg/kg, i.p., qod, respectively). Quantified expression levels measured as integrated optical density per area. Compared to control, HA15 significantly increased the immunoreactivity of HSPA5 (0.11 vs. 0.17), Ki-67 (0.24 vs. 0.38), and MMP9 (0.17 vs. 0.27), while HA15 + 4μ8C combined therapy significantly increased that of HSPA5 (0.11 vs. 0.15), ATF5 (0.11 vs. 0.24), DDIT3 (0.11 vs. 0.29), and c-CASP3 (0.10 vs. 0.22). Compared to HA15 monotherapy, HA15 + 4μ8C combined therapy significantly increased immunoreactivity of ATF5 (0.12 vs. 0.24), DDIT3 (0.16 vs. 0.29) and c-CASP3 (0.09 vs. 0.22), while it reduced that of Ki-67 (0.38 vs. 0.18), and MMP9 (0.27 vs. 0.18). Statistical significance is denoted by asterisks (* $P < 0.05$, ** $P < 0.01$, *** $P < 0.001$; one-way ANOVA test). Abbreviations: ATF5, activating transcription factor 5; c-CASP3, cleaved caspase 3; DDIT3, DNA damage-inducible transcript 3; HCHS, high-grade chondrosarcoma; HSPA5, heat shock protein family A member 5; i.p., intraperitoneally; Ki-67, marker of proliferation Ki-67; MMP9, matrix metallopeptidase 9; qod, every other day.

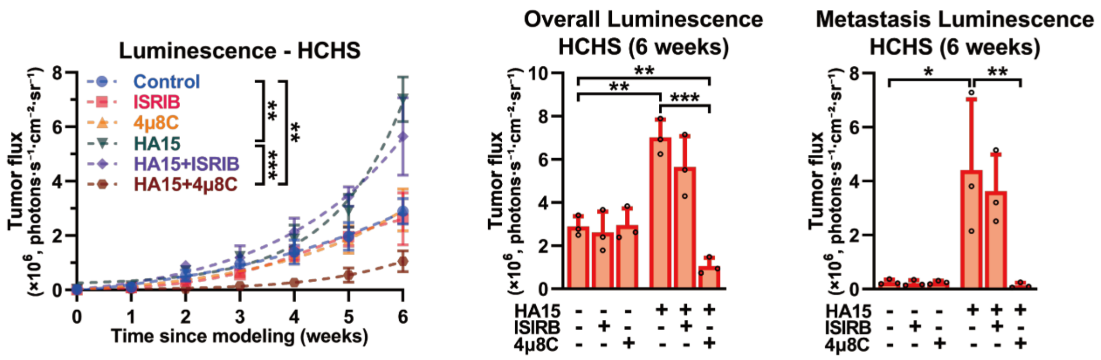

Supplementary Figure S13. Real-time and endpoint bioluminescence of high-grade chondrosarcoma (HCHS) tumor burden over a 6-week treatment period. The left panel shows real-time bioluminescence of HCHS tumor burden over 6 weeks, the middle panel displays the endpoint quantification, and the right panel presents metastasis luminescence, all comparing treatments of control, HA15 (20 mg/kg, i.p., qod), 4μ8C (20 mg/kg, i.p., qod), and HA15 + 4μ8C (20 + 20 mg/kg, i.p., qod, respectively). Quantification of tumor flux is presented as photons·s^{-1}·cm^{-2}·sr^{-1} (×10^{6}). Compared to control, treatment with HA15 alone resulted in a significant, approximately 2.4-fold increase in overall bioluminescent signal (from 2.89 to 7.02) and 17.0-fold increase in metastasis bioluminescent signal (from 0.26 to 4.41). Compared to HA15 monotherapy, combined treatment with HA15 and 4μ8C led to a significant, approximately 6.7-fold reduction in overall signal (from 7.02 to 1.05) and 33.9-fold reduction in metastasis bioluminescent signal (from 4.41 to 0.13). Statistical significance is denoted by asterisks (* $p < 0.05$, ** $p < 0.01$, *** $p < 0.001$; one-way ANOVA test). Abbreviations: HCHS, high-grade chondrosarcoma; i.p., intraperitoneally; qod, every other day.

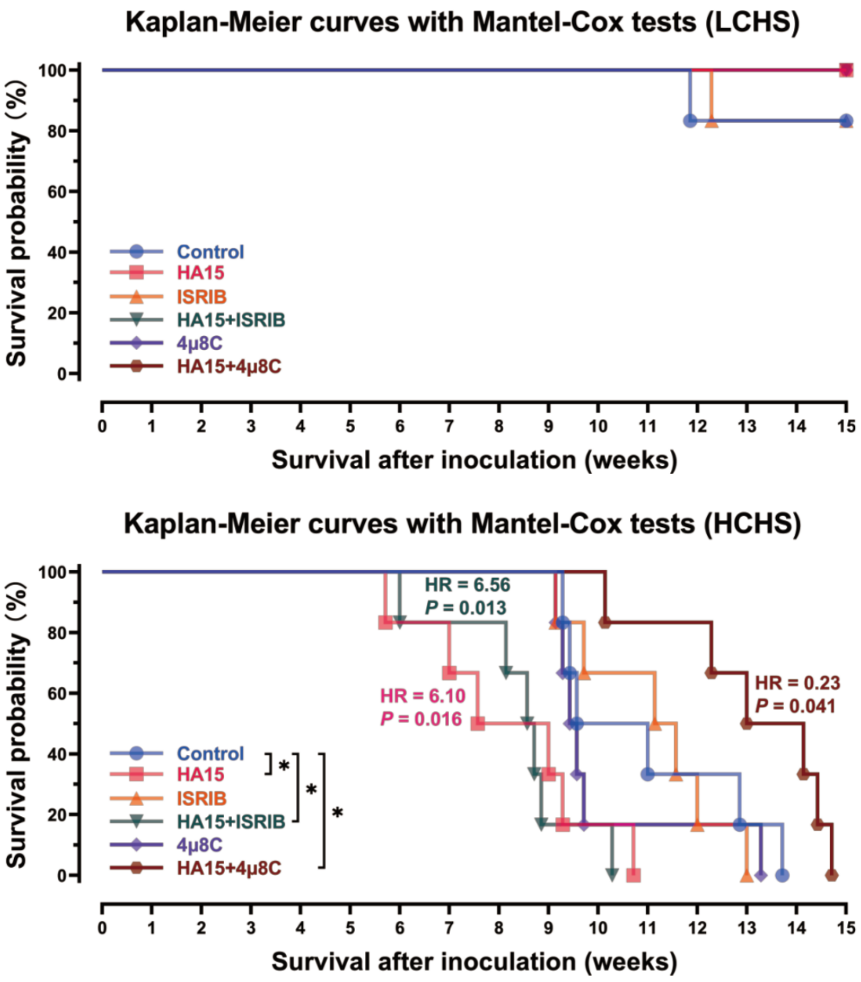

Supplementary Figure S14. Kaplan-Meier curves of LCHS (up panel) and HCHS (bottom panel) over 15 weeks with treatments of control, HA15 (20 mg/kg, i.p., qod), 4μ8C (20 mg/kg, i.p., qod), HA15 + 4μ8C (20 + 20 mg/kg, i.p., qod, respectively). In LCHS, low mortality was observed in all groups. In HCHS, compared to control, HA15 monotherapy reduced the median survival from 9.57 to 7.57 weeks with a hazard ratio of 6.10, while the combined therapy with HA15 and 4μ8C extended the median survival from 9.57 to 13.00 weeks with a hazard ratio of 0.23. Asterisks (*) indicate statistically significant differences (*P* < 0.05, Mantel-Cox test). Abbreviations: HCHS, high-grade chondrosarcoma; HR, hazard ratio; i.p., intraperitoneally; qod, every other day.
